# Pulmonary Tumor Embolism Complicated by Metastatic Liver Carcinoma in Female With Primary Breast Carcinoma

**DOI:** 10.7759/cureus.37416

**Published:** 2023-04-11

**Authors:** Zanib Shahbaz, Pugazhendi Inban, Dev K Patel, Tass Sayeed, Baris Tarimci, Idowu O Adewole, Nabi Nadia, Prashant Obed R Dundi, Taha Sajjad, Aadil Khan

**Affiliations:** 1 Department of Research and Development, Windsor University School of Medicine, Chicago, USA; 2 Department of General Medicine, Government Medical College, Chennai, IND; 3 Department of Internal Medicine, Smt. Nathiba Hargovandas Lakhmichand (NHL) Municipal Medical College, Ahmedabad, IND; 4 Department of Medicine, Windsor University School of Medicine, Chicago, USA; 5 Department of Internal Medicine, Ege University Faculty of Medicine, Izmir, TUR; 6 Department of Medicine, All Saints University School of Medicine, Roseau, DMA; 7 Department of Obstetrics and Gynecology, Government Medical College, Srinagar, IND; 8 Department of General Practice, Karnataka Institute of Medical Sciences, Bangalore, IND; 9 Department of Medical Education, Mountain Vista Medical Center (MVMC), Phoenix, USA; 10 Department of Internal Medicine, Lala Lajpat Rai (LLR) Hospital, Kanpur, IND

**Keywords:** metastases, chemotherapy, hepatic tumors, pulmonary embolus, liver

## Abstract

Pulmonary tumor embolism (PTE) is a rare phenomenon typically presenting as dyspnea in cancer patients. Primary pathophysiology is similar to the thromboembolic disease of the pulmonary vasculature, which involves large vessels to small arterioles. This phenomenon occurs mostly in lung, stomach, liver, and breast adenocarcinoma. The symptoms of hypoxemia and the signs of hemodynamic instability and high-resolution computed tomography (CT) scans, along with a histopathological examination, are essential to make a confirmatory diagnosis of pulmonary tumor embolism. However, treatment options to effectively treat pulmonary tumor embolus are limited and still under investigation. We present a rare case of pulmonary tumor embolism in a patient with metastatic liver carcinoma and its management in a female with primary breast carcinoma.

## Introduction

Pulmonary tumor embolism (PTE) is an unusual form of metastasis to the lungs associated with respiratory and hemodynamic compromise in cancer patients. The tumor cells occlude pulmonary microvasculature and present a clinical presentation similar to thromboembolic disease [1]. Furthermore, the occluding tumor emboli can further activate the coagulation system leading to concomitant obstruction in other parts of the body. It ranges from large masses similar to the classical pulmonary embolism (PE) to microvessel emboli occluding small arterioles causing subacute clinical syndrome [2]. The incidence of tumor embolism is about 3%-26% for solid tumors, estimated by a case series of autopsies. Tumor embolisms are considered to be more common in mucin-producing adenocarcinomas such as gastric, lung, and breast cancer [3]. The staging of primary tumors such as liver, breast, stomach, kidney, and prostate carcinoma can assess the risk of developing pulmonary tumor embolism, justifying the higher risk of embolism in advanced-stage cancer. Generally, clinicians correlate the risk of pulmonary tumor embolism with advanced-stage tumors that usually metastasize to the lungs [4]. PE along with liver metastatic carcinoma is uncommon, and a recent study identified that out of 658 autopsies, 20 patients had pulmonary tumor emboli. It was identified that all the patients had advanced carcinoma with distant metastases, and metastatic liver carcinoma was observed in 14 patients. It was also reported that breast carcinoma was among the most common primary malignancy overall [5]. This case study includes an elderly female patient with breast carcinoma with secondary metastasis to the liver and pulmonary embolus tumor.

## Case presentation

A 71-year-old female with a past medical history of breast carcinoma, cholelithiasis, hypertension, and secondary metastasis to the liver presented to the outpatient department complaining of chest pain, breathlessness, and swelling of the left lower limb over the last 10 days. There were no symptoms of fever or cough. She had no alcohol, tobacco, and smoking history. She was not compliant with her medications. She underwent a bilateral mastectomy three years ago and refused chemotherapy two years back.

On physical examination, she had tachycardia (109/minute), hypoxemia (90%), and mild elevation in blood pressure (150/100 mmHg) with features of rapid-onset cor pulmonale, including distended jugular veins, lower extremity pitting edema, and parasternal systolic lift. She was well-oriented to time, place, and person. On breast examination, there was a surgical scar mark with no palpable lump, and mild tenderness was present on the left upper quadrant of the left breast. Bilateral axillary lymph nodes were palpable. Her chest ultrasonography was unremarkable. The rest of the systemic examination was unremarkable. Her electrocardiogram (EKG) was suggestive of anterolateral ischemia, for which morphine and nitrates were used. Her troponin I test was negative, and her EKG remained unchanged on the serial EKGs. Her chest X-ray was unremarkable. An initial arterial blood gas analysis revealed arterial carbon dioxide tension of 31 mmHg and arterial oxygen tension of 59 mmHg. Her laboratory results showed an increased level of D-dimer (31 ug/ml) and pro-brain natriuretic peptide (BNP) (799 pg/ml).

**Figure 1:**
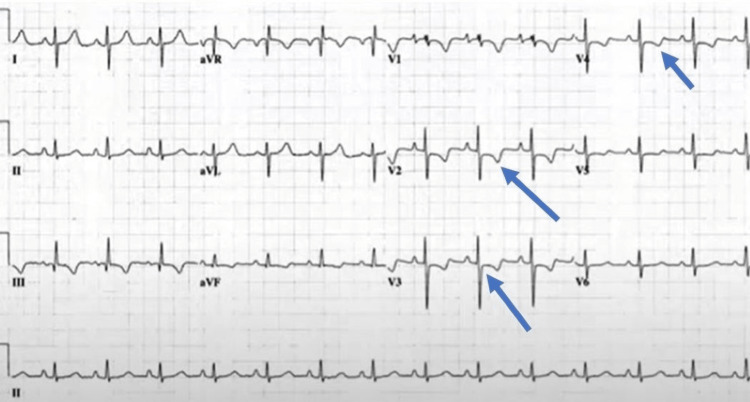
Echocardiogram demonstrating T wave inversion in anterior leads.

**Figure 2:**
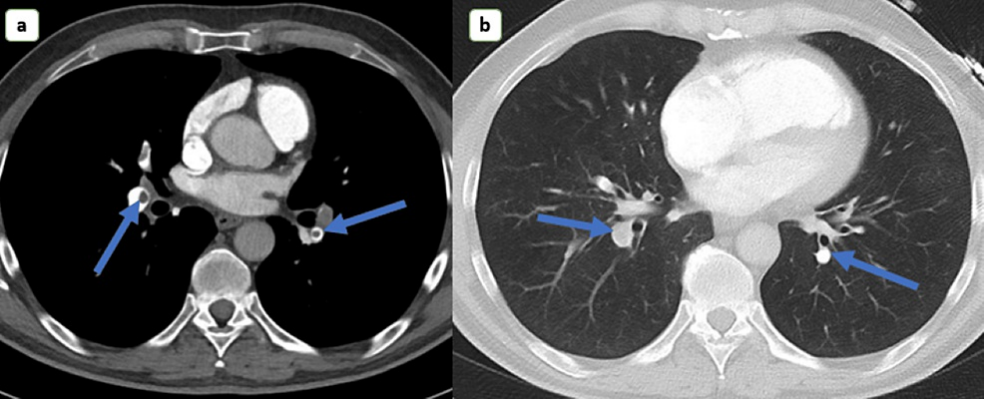
Computed tomography demonstrating emboli in both right and left main pulmonary arteries and their associated branches.

The following day, her dyspnea and chest pain worsened. A repeat EKG revealed T wave inversion on anterior leads, raising suspicion of pulmonary embolism (Figure 1). On chest computed tomography (CT), fibrocalcific lesions were seen in bilateral lung fields, predominantly in the right upper, with associated cicatricial emphysema and no mediastinal lymphadenopathy. There were enlarged left axillary lymph nodes. On CT angiography, emboli were observed in the right and left main pulmonary arteries and their associated branches (Figure 2). A transbronchial lung biopsy revealed malignant cells in the pulmonary artery and confirmed a diagnosis of pulmonary tumor embolism.

**Figure 3:**
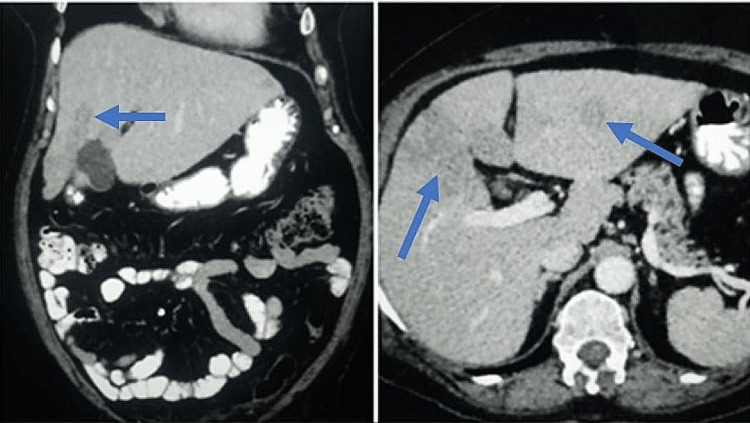
Abdominal computed tomography demonstrating hepatomegaly with mild hypoattenuation of liver parenchyma and multiple variable-sized peripherally enhancing hypodense lesions in both lobes of the liver.

Contrast-enhanced CT scan of the whole abdomen was performed, which showed hepatomegaly with mild hypoattenuation of liver parenchyma and a few variable-sized peripherally enhancing hypodense lesions in both lobes of the liver and elevated alpha-fetoprotein (533 ng/ml) (Figure 3). A microscopic examination of the liver lesion revealed prominent sheets, clusters of reactive hepatocytes, and a few singly lying malignant cells. These malignant cells had pleomorphic round to oval nuclei, coarsely clumped chromatin, occasional prominent nucleoli, scant cytoplasm, and stripped nuclei on a hemorrhagic background mixed with necrosis most likely suggestive of metastatic liver carcinoma from breast carcinoma.

Her diagnosis was confirmed to be pulmonary tumor thromboembolism and multiple space-occupying liver lesions due to metastasis from breast carcinoma. She was treated with rivaroxaban, which was later changed to argatroban. Further treatment was planned with doxorubicin and cisplatin chemotherapy for the metastatic carcinoma and was kept under follow-up.

## Discussion

The pulmonary tumor embolism occurrence rate is 3%-26% among solid tumor patients. It is a critical pulmonary disorder in less than 1% of cancer patients [3]. Some cases can develop liver failure before symptomatic pulmonary tumor embolism. Since pulmonary tumor embolism is the most challenging to diagnose because of its low incidence, it is usually diagnosed after death. In a case series, some investigators performed an autopsy, including radiography for tumor embolism, and confirmed pulmonary tumor embolism as a cause of death with a favorable ratio of one out of eight patients [6]. Some investigators suggest that interstitial infiltrates on chest CT are more likely associated with a lymphatic disease, which is a distinguishing element between tumor emboli and carcinomatosis [7]. Pulmonary hypertension may also be evident on echocardiography; in our case, we also observed the cor pulmonale; however, echocardiography was not performed. The severity of the patient's symptoms does not depict the degree of vascular obstruction. Right heart catheterization and ventilation-perfusion scanning methods are good diagnostic tools for diagnosing microscopic pulmonary tumor emboli [5]. These methods are beneficial in developing appropriate therapy for cancer patients and preventing the use of unnecessary anticoagulation therapies.

The pathophysiology of PTE is complex and multifactorial, involving a number of different processes that contribute to the formation of tumor emboli in the pulmonary vasculature. The first step in the development of PTE is the entry of tumor cells into circulation. This can occur through various mechanisms, including direct invasion of tumor cells into the blood vessels, shedding tumor cells from the primary tumor site to the lymphatic system, or disseminating tumor cells into the bloodstream via tumor-associated neovasculature [6,8]. Once tumor cells are in circulation, they can be transported throughout the body, including to the lungs. Once in the pulmonary circulation, tumor cells must adhere to the endothelial cells lining the blood vessels to migrate into the vessel wall and form emboli. This process is mediated by various adhesion molecules, including selectins, integrins, and cadherins [9]. Tumor cells also secrete various pro-inflammatory cytokines and chemokines, which can further promote the adhesion and migration of tumor cells into the vessel wall. As tumor cells migrate into the vessel wall, they can form small aggregates that gradually increase in size over time. These aggregates can eventually form larger tumor emboli, obstructing blood flow in the pulmonary vasculature [6-9]. The diagnosis of PTE is often challenging, as the symptoms can be nonspecific and mimic those of other conditions, such as pulmonary embolism or pneumonia. Imaging studies such as CT, magnetic resonance imaging (MRI), and positron emission tomography (PET) scans can help confirm the diagnosis and identify the primary tumor [10].

The management of PTE depends on several factors, including the size and location of the emboli, the extent of the primary tumor, and the patient's overall health status. In general, treatment options for PTE can be divided into three categories: systemic therapy, interventional therapy, and supportive care [11]. Systemic therapy involves chemotherapy or targeted therapy to shrink the primary tumor and prevent the formation of new emboli. In some cases, systemic therapy may also be used to target the emboli in the lungs directly [12]. The choice of systemic therapy depends on the type of cancer and the patient's overall health status [6]. Chemotherapy is typically the first-line treatment for most types of cancer, but targeted therapy may be used in some cases if the tumor has specific genetic mutations. Interventional therapy involves using minimally invasive procedures to remove or break up the tumor emboli. These procedures include catheter-directed thrombolysis, where a catheter is inserted into the lungs and a clot-dissolving drug is injected directly into the emboli, and percutaneous mechanical thrombectomy, where a catheter with a mechanical device is used to break up the emboli [12]. Interventional therapy is typically reserved for patients who are not candidates for or have failed systemic therapy. Supportive care is an essential component of the management of PTE. Patients with PTE may experience shortness of breath, chest pain, and other symptoms that require management. Oxygen therapy, pain management, and anticoagulation therapy may alleviate symptoms and prevent further complications. Anticoagulation with unfractionated heparin is recommended in tumor-associated PE for at least six months. Patients with active malignancy or ongoing cancer therapy are the strongest candidate for long-term anticoagulation [13]. In addition, patients with PTE require close monitoring for the development of other complications, such as pulmonary hypertension or right-sided heart failure [14]. The prognosis of PTE varies depending on several factors, including the extent of the emboli, the type and stage of the primary tumor, and the patient's overall health status.

## Conclusions

PTE is an uncommon presentation of malignant tumor associated with high morbidity and mortality. Early therapeutic intervention with anticoagulation, adjuvant chemotherapy, and supportive therapy are potentially curative approaches. In this case, we thoroughly investigated and managed the patient's pulmonary tumor embolism complicated by metastatic liver carcinoma from primary breast carcinoma. PTE should be among differential diagnoses in patients presenting with dyspnea and chest pain with underlying malignancy.
